# Editorial: Deep learning for disease prediction in next-generation sequencing and biomedical imaging data

**DOI:** 10.3389/fgene.2023.1260940

**Published:** 2023-08-04

**Authors:** Saurav Mallik, Junichi Iwata, Ruifeng Hu, Tapas Si

**Affiliations:** ^1^ Department of Environmental Health, Harvard T. H. Chan School of Public Health, Boston, MA, United States; ^2^ School of Dentistry, The University of Texas Health Science Center at Houston, Houston, TX, United States; ^3^ Department of Neurology, Brigham and Women’s Hospital and Harvard Medical School Boston, Boston, MA, United States; ^4^ Department of Computer Science and Engineering, University of Engineering and Management, Jaipur, Gurukul, Jaipur, Rajasthan, India

**Keywords:** deep learning, machine learning, next-generation sequencing, biomedical imaging, optimization algorithm

Computational learning, especially deep learning and machine learning, has had a huge impact. This Research Topic gathered articles on these two fundamental concepts which show how deep learning and machine learning approaches have been applied to array-based biomedical data such as next-generation sequencing (NGS) and medical imaging data.

Overall, our Research Topic published 11 articles of which 8 covered research on array-based data, while the remaining 3 articles belonged to studies on biomedical imaging. Among them, She et al. propose a joint mathematical model integrating a random forest classifier and artificial neural network (ANN) for the possible diagnosis of the estrogen-dependent inflammatory disease endometriosis. The method utilizes publicly available gene expression datasets in the Gene Expression Omnibus (GEO) and estimated seven significant differentially expressed genes (DEGs) (viz., COMT, NAA16, CCDC22, EIF3E, AHI1, DMXL2, and CISD3) through the random forest classifier, while three of them (AHI1, DMXL2, and CISD3) were novel signatures useful for the pathogenesis of endometriosis. Related KEGG pathway and GENE Ontology analysis is also performed to obtain the biological significance of the signatures. Niu et al. conduct a comprehensive bioinformatic analysis to determine the potential diagnostic and prognostic genetic markers for gastric cancer. In this study, several markers (COL1A1, COL5A2, P4HA3, and SPARC) yielded high scores in the prognosis and diagnosis of gastric cancer, hence they are named as the respective diagnosis and prognosis markers for the disease. A second study conducted by the same team Niu et al. focuses on an extracellular matrix protein, prolyl 4-hydroxylase subunit alpha 3 (P4HA3), and thus performed an extensive protein-protein interaction and prognosis analysis in terms of correlating it with immune infiltration in the gastric Cancer. Another study was conducted by Gu et al. in which an angiogenic factor-based gene signature is identified that had a significant response in patients’ survival, disease prognosis, and immunotherapy in non-small-cell lung cancer, a common malignancy. The corresponding model had good discrimination and calibration and may predict the disease prognosis of treatment in the respective clinical practice. Wei et al. provide a comprehensive bioinformatic analysis to determine a potential prognostic genetic marker (viz., GNG7) for the lung adenocarcinoma that correlates with the immune infiltrates. Wen et al. introduce a framework by integrating several machine learning algorithms to determine whether hub genes are useful for the diagnosis of ankylosing spondylitis by validating several respective gene expression datasets. A novel machine learning and optimization framework termed as 3-factor penalized non-negative matrix factorization-based multiple kernel learning with the soft margin hinge loss (3PNMF-MKL) is proposed by Mallik et al. where two consecutive steps, namely, multi-modal data integration and gene signature discovery are conducted.

Essential genes are required for critical cellular activities in the overall survival of many species. Rout et al. conduct an extensive analysis to determine the discriminant features (genes) from the stationary pattern of the nucleotide bases (A, T, G, C) and their respective application towards the classification of the essential gene.

From the imaging point of view, a dual-input convolution neural network (CNN) with the local interpretable model-agnostic explanation (LIME) and Shapley additive explanation (SHAP) is utilized to predict the discrete subtypes of brain tumors, viz., glioma, meningioma, and pituitary through the Magnetic Resonance Imaging (MRI) of brain (Gaur et al.). Another study was conducted by Sharma et al. where the likelihood of a colorectal cancer patient dying could be significantly decreased through the early diagnosis as well as treatment of the pre-cancerous polyps. Sharma et al. develop an ensemble-based deep CNN model that helped to identify the polyps from a colonoscopy video with a higher accuracy which outperformed the existing methodologies (viz., ResNet101, Xception, and GoogleNet). The projections of the lateral chest radiograph (chest X-rays or, CXR) of children with clinically suspected pulmonary tuberculosis (TB) yielded a significant enhancement in the overall sensitivity of the enlarged lymph nodes. A model-level ensemble was built through the fine-tuned CNN and Vision Transformers (ViT) models by Rajaraman et al. to detect the TB-consistent outcomes in the lateral CXRs, and finally, a significantly better classification performance could be obtained.

This Research Topic covers articles on developing frameworks/tools/algorithms for handling next-generation sequencing (NGS) array-based data as well as medical imaging data. It is expected that future machine/deep learning software will be increasingly helpful for biomedical and healthcare researchers to realize the utilization of machine/deep learning and optimization to improve the overall research quality and integrity in disease diagnosis and potential therapeutic use.

